# Carotenoids in Staple Cereals: Metabolism, Regulation, and Genetic Manipulation

**DOI:** 10.3389/fpls.2016.01197

**Published:** 2016-08-10

**Authors:** Shengnan Zhai, Xianchun Xia, Zhonghu He

**Affiliations:** ^1^National Wheat Improvement Center, Institute of Crop Science, Chinese Academy of Agricultural SciencesBeijing, China; ^2^International Maize and Wheat Improvement Center, Chinese Academy of Agricultural SciencesBeijing, China

**Keywords:** carotenoid metabolism, carotenoid regulation, marker-assisted breeding, metabolic engineering, provitamin A biofortification, *Triticum*

## Abstract

Carotenoids play a critical role in animal and human health. Animals and humans are unable to synthesize carotenoids *de novo*, and therefore rely upon diet as sources of these compounds. However, major staple cereals often contain only small amounts of carotenoids in their grains. Consequently, there is considerable interest in genetic manipulation of carotenoid content in cereal grain. In this review, we focus on carotenoid metabolism and regulation in non-green plant tissues, as well as genetic manipulation in staple cereals such as rice, maize, and wheat. Significant progress has been made in three aspects: (1) seven carotenogenes play vital roles in carotenoid regulation in non-green plant tissues, including 1-deoxyxylulose-5-phosphate synthase influencing isoprenoid precursor supply, phytoene synthase, β-cyclase, and ε-cyclase controlling biosynthesis, 1-hydroxy-2-methyl-2-(*E*)-butenyl 4-diphosphate reductase and carotenoid cleavage dioxygenases responsible for degradation, and orange gene conditioning sequestration sink; (2) provitamin A-biofortified crops, such as rice and maize, were developed by either metabolic engineering or marker-assisted breeding; (3) quantitative trait loci for carotenoid content on chromosomes 3B, 7A, and 7B were consistently identified, eight carotenogenes including 23 loci were detected, and 10 gene-specific markers for carotenoid accumulation were developed and applied in wheat improvement. A comprehensive and deeper understanding of the regulatory mechanisms of carotenoid metabolism in crops will be beneficial in improving our precision in improving carotenoid contents. Genomic selection and gene editing are emerging as transformative technologies for provitamin A biofortification.

## Introduction

Carotenoids are mainly C40 isoprenoids comprising a large family with more than 700 members that are widely distributed in plants, algae, fungi, and bacteria (Khoo et al., 2011). In plants, they perform a multitude of functions involving the photosynthetic apparatus, photoprotection, and precursors to phytohormones such as ABA and strigolactones (Niyogi, 2000; Cazzonelli and Pogson, 2010). In addition, carotenoids provide color and aroma to flowers and fruits for attracting insects and other organisms for pollination and seed dispersal, and protect the seed from deterioration (Walter et al., 2010; Moise et al., 2013). Very recently, carotenoid derivatives were found in association with response to environmental stresses, such as photoxidative stress (Havaux, 2014).

Carotenoids also play a critical role in animal and human health. In animals, they can improve sexual behavior and reproduction, and protect animals from predation as well as parasitism (McGraw and Toomey, 2010). For humans, the most important function of carotenoids is as a dietary source of provitamin A (mainly α-carotene, β-carotene, zeaxanthin, and β-cryptoxanthin; Giuliano et al., 2008). Vitamin A deficiency (VAD) is the leading cause of preventable blindness in children and increases the risk of disease and death from severe infections. For pregnant women, VAD may cause night blindness and increase the risk of maternal mortality. The World Health Organization has estimated that 250,000–500,000 vitamin A-deficient children became blind each year, with half of them dying from loss of eyesight within 12 months^1^ In addition, carotenoids as antioxidants have a protective function in reducing the risk of age-related macular degeneration (ARMD), cancer, cardiovascular diseases, and other chronic diseases (Fraser and Bramley, 2004). Carotenoids are also used commercially as feed additives to enhance pigmentation of fish and eggs, colorizing agents for human food, cosmetics, and pharmaceutical products (Sandmann, 2001). Thus, understanding the regulatory mechanisms of carotenoids is a very important scientific pursuit and biofortification of staple foodstuffs for health benefits has become an important issue in food production.

Because animals and humans are unable to synthesize carotenoids *de novo* they rely upon diet as the source of these compounds. However, most staple cereals, such as rice (*Oryza sativa*), wheat (*Triticum aestivum*), and maize (*Zea mays*), contain very little amounts of carotenoids in their grains. Therefore, the genetic manipulation of carotenoid accumulation in staple cereal grains should be a powerful means to combat vitamin A deficiency, and especially important for developing countries where people frequently rely on a single crop for sustenance. For better genetic manipulation of carotenoid content within cereal grains there is a particular interest in the regulatory mechanisms of carotenoid biosynthesis in non-green plant tissues (Farré et al., 2011). Various lines of evidence show that key nodes in the MEP pathway, carotenoid metabolism, and sequestration sink play vital roles in regulation of carotenoid biosynthesis.

In this review, we focus on carotenoid metabolism and regulation in non-green plant tissues, as well as genetic manipulation in staple cereals including rice, maize, and wheat. Compared with maize and rice (Harjes et al., 2008; Yan et al., 2010; Breitenbach et al., 2014; Bai et al., 2016), carotenoid biosynthesis in wheat has received much less attention. Therefore, a comprehensive overview of carotenoid biosynthesis in wheat was undertaken to provide a platform of understanding of carotenoid biosynthesis as wheat supplies significant amounts of dietary carbohydrate and protein for over 60% of the world population, and is also an important source of carotenoids in human diets (Shewry, 2009). In addition to cereals, the extensive literature on carotenoid biosynthesis in bacteria or other plants is also discussed, as it contributes to a better understanding of the pathway in cereals.

## Carotenoid Metabolism

**FIGURE 1:**
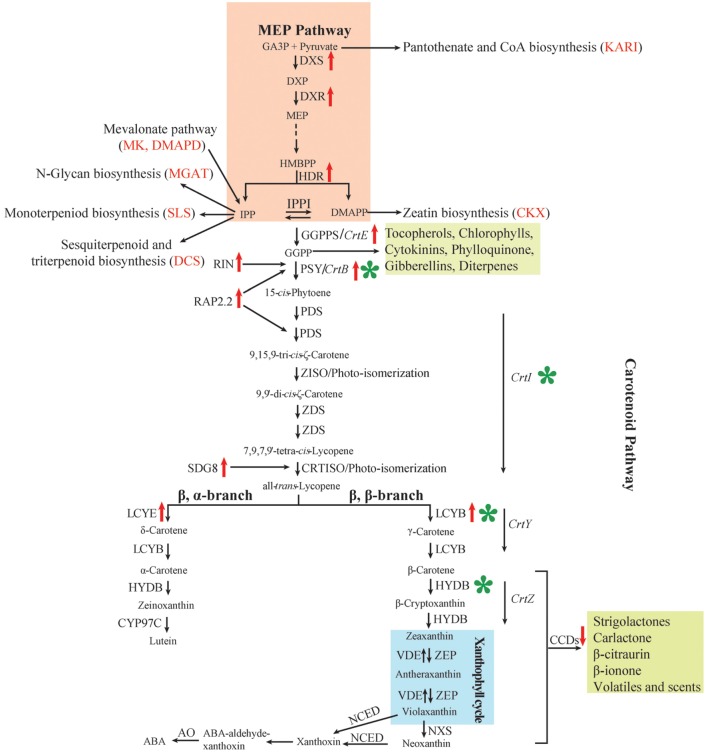
**Carotenoid metabolism, regulation and genetic manipulation in higher plants.** Names of bacterial enzymes are in italics. Candidate genes for carotenoid accumulation obtained by QTL analysis are displayed in parentheses and in red. Red upward pointing arrows, gene expression positively correlated with carotenoid biosynthesis; red downward pointing arrows, gene expression negatively correlated with carotenoid biosynthesis; green asterisk, main genetic manipulation nodes in staple cereals. Other MEP isoprenoid-derived metabolites and carotenoid cleavage products apocarotenoids are shown in the green box. ABA, abscisic acid; AO, aldehyde oxidase; CCD, carotenoid cleavage dioxygenase; CKX, cytokinin oxidase/dehydrogenase; CrtB, bacterial phytoene synthase; CrtE, bacterial GGPP synthase; CrtI, bacterial phytoene desaturase/isomerase; CRTISO, carotene isomerase; CrtY, bacterial lycopene β-cyclase; CrtZ, bacterial β-carotene hydroxylase; CYP97C, heme-containing cytochrome P450 carotene ε-ring hydroxylase; DCS, delta-cadinene synthase; DMADP, dimethylallyl diphosphate; DXP, 1-deoxy-D-xylulose 5-phosphate; DXR, 1-deoxy-D-xylulose 5-phosphate reductoisomerase; DXS, 1-deoxyxylulose-5-phosphate synthase; GA3P, D-glyceraldehyde-3-phosphate; GGPP, geranylgeranyl diphosphate; GGPPS, GGPP synthase; HDR, 1-hydroxy-2-methyl-2-(*E*)-butenyl 4-diphosphate reductase; HYDB, β-carotene hydroxylase [also known as non-heme di-iron β-carotene hydroxylase (BCH) and heme-containing cytochrome P450 β-ring hydroxylase (CYP97A and CYP97B)]; IPP, isopentenyl diphosphate; IPPI, IPP isomerase; KARI, ketol-acid reductoisomerase; LCYB, lycopene β-cyclase; LCYE, lycopene ε-cyclase; MEP, 2C-methyl-D-erythritol-4-phosphate; MGAT1, alpha-1, 3-mannosyl-glycoprotein 2-beta-*N*-acetylglucosaminyltransferase; MK, mevalonate kinase; NCED, 9-*cis*-epoxycarotenoid dioxygenase; NXS, neoxanthin synthase; PDS, phytoene desaturase; PSY, phytoene synthase; RAP2.2, a member of the APETALA2 (AP2)/ethylene-responsive element-binding protein transcription factor family; RIN, MADS-box transcription factor RIPENING INHIBITOR; SDG8, SET2 histone methyltransferase; SLC, secologanin synthase; VDE, violaxanthin de-epoxidase; ZDS, ζ-carotene desaturase; ZEP, zeaxanthin epoxidase; ZISO, ζ-carotene isomerase.

Carotenoid metabolism in plants is a complex process, and has been extensively characterized in a range of organisms providing an almost complete pathway for carotenogenesis and degradation (Cunningham and Gantt, 1998; Giuliano et al., 2008). The main steps of carotenoid metabolism in higher plants are briefly summarized below and presented in **Figure 1.**

### Biosynthesis

Carotenoids are derived from the plastid-localized MEP pathway for which glyceraldehyde-3-phosphate and pyruvate act as initial substrates leading to the synthesis of GGPP, the common precursor for biosynthesis of carotenoids and several other terpenoid compounds (Farré et al., 2010; Rodriguez-Concepcion, 2010). The first committed step in the carotenoid biosynthesis pathway is condensation of two GGPP molecules by PSY to produce 15-*cis*-phytoene. Phytoene is converted into lycopene by two desaturation reactions catalyzed by PDS and ZDS. These enzymes give rise to poly-*cis* compounds which are converted to the all-*trans* form by ζ-carotene isomerase (ZISO) and CRTISO, as well as a light-mediated photo-isomerization. In bacteria, a single enzyme, CrtI, is believed to confer the same desaturation and isomerization reactions.

Lycopene constitutes a branching point in the pathway since it is the substrate of two competing cyclases, LCYB and LCYE. α-carotene is produced when LCYE and LCYB act together on the two ends of lycopene (β, ε-branch), whereas β-carotene is formed when LCYB acts alone (β, β-branch). Alpha-carotene and β-carotene are hydroxylated to produce lutein and zeaxanthin, respectively. These reactions are catalyzed by the β-ring carotene hydroxylase [HYDB, also known as non-heme di-iron β-carotene hydroxylase (BCH) or heme-containing cytochrome P450 β-ring hydroxylase (CYP97A and CYP97B)] and heme-containing cytochrome P450 carotene ε-ring carotene hydroxylase (CYP93C). Whereas lutein represents the natural end point of the β, ε-branch, zeaxanthin is further epoxidized by zeaxanthin epoxidase (ZEP) in a two-step reaction to produce violaxanthin via antheraxanthin. This reaction is reversed by violaxanthin deepoxidase (VDE) to give rise to the xanthophyll cycle for plants to adapt high light stress (Demmig-Adams and Adams, 2002). Violaxanthin is converted into neoxanthin by neoxanthin synthase (NXS), the final carotenoid of the β, β-branch of the classical biosynthetic pathway.

In some plants, the classical carotenoid biosynthesis pathway extends further to synthesize specialized ketocarotenoids. One such example is the red fruits of chili peppers, where the capsanthin and capsorubin are synthesized from antheraxanthin and violaxanthin by capsanthin-capsorubin synthase (CCS) enzyme (Gómez-García and Ochoa-Alejo, 2013). Another example is the ornamental plant *Adonis aestivalis* whose petals synthesize the red ketocarotenoid astaxanthin, which is usually found in microbes (Cunningham and Gantt, 2005). With progress in high-performance liquid chromatography-tandem mass spectrometric (HPLC-MS) and high-performance liquid chromatography-nuclear magnetic resonance (HPLC-NMR) technologies, more specialized ketocarotenoids will be detected, which will further enrich our knowledge of this pathway.

In grasses, PSY are encoded by three paralogous genes (*PSY*1-3; Dibari et al., 2012). *PSY1* is correlated with carotenoid accumulation in grain, *PSY2* is involved in protecting the photosynthetic apparatus from photo-oxidative degradation in green tissues, and *PSY3* is associated with root carotenogensis channeled into ABA formation, mainly responsing to abiotic stresses, such as drought and salt (Gallagher et al., 2004; Li et al., 2008; Welsch et al., 2008). *PSY* duplication has provided an opportunity for subfunctionalization whereby gene family members vary in tissue specificity of expression to control carotenogenesis independently of photosynthesis or in response to certain stresses (Li et al., 2008; Welsch et al., 2008; Arango et al., 2010).

### Degradation

Carotenoid degradation can occur via non-specific mechanisms such as photo chemical oxidation or LOX (Siedow, 1991; Auldridge et al., 2006). However, specific tailoring of carotenoids is carried out by a family of CCDs, which appear to have different substrate preferences (Vallabhaneni and Wurtzel, 2009). The CCD gene family is divided into two types: nine-*cis*-epoxycarotenoid dioxygenases (NCEDs) catalyze both violaxanthin and neoxanthin to produce xanthoxin, the precursor of ABA (Seo and Koshiba, 2002; Walter et al., 2010), and CCDs that catalyze a vast array of different cleavage steps giving rise to apocarotenoids. For example, CCD1 is involved in β-ionone biosynthesis, whereas CCD7 and CCD8 are associated with strigolactone biosynthesis. These apocarotenoids are crucial for various biological processes in plants, such as regulation of growth and development and plant-insect interaction (Walter et al., 2010; Alder et al., 2012; Avendano-Vazquez et al., 2014).

### Sequestration

Carotenoids are usually synthesized *de novo* in nearly all types of differentiated plastids of leaves, roots, flowers, fruits, and seeds, including chloroplasts, chromoplasts, amyloplasts, elaioplasts, leucoplasts, and etioplasts, but accumulate in large quantities in chloroplasts and chromoplasts (Howitt and Pogson, 2006; Cazzonelli and Pogson, 2010). Chloroplasts and chromoplasts differ considerably in the way they sequestrate end-product carotenoids. In chloroplasts, carotenoids are located in photosynthetic membranes and integrated with chlorophyll-binding proteins to form pigment–protein complexes (Vishnevetsky et al., 1999). Whereas, in chromoplasts, carotenoids are associated with polar lipids and carotenoid associated proteins to form carotenoid-lipoprotein sequestering substructures (e.g., globules, crystals, membranes, fibrils, and tubules) to effectively sequester and retain a large quantity of carotenoids (Vishnevetsky et al., 1999; Egea et al., 2010; Li and Yuan, 2013).

To date, there is little understanding of carotenoid degradation. Much more effort to understand CCD gene family members, their substrates and products, is still needed. In addition, some acronyms of carotenogenes were confused in the previous literature, such as β-hydroxylases being replaced by BCH and HYD in rice (Du et al., 2010), crtRB1 and HYD in maize (Yan et al., 2010), BCH in *Arabidopsis* (Kim et al., 2009), and CHY in potato (Diretto et al., 2007), respectively. For a better understanding and communication, international efforts are needed to uniform the acronyms.

## Carotenoid Regulatory Mechanisms in Non-Green Plant Tissues

Relatively little is known about the regulation of carotenogenesis in chloroplasts. Although expression of carotenoid genes does take place in etiolated plants, most carotenoid biosynthetic genes, including those in the MEP pathway, are activated during light-triggered de-etiolation (Giuliano et al., 2008; Cazzonelli and Pogson, 2010; Rodriguez-Concepcion, 2010). The phytochrome-interacting factor 1 (PIF1) is shown to bind to the *PSY* promoter and represses *PSY* expression under dark conditions. Toledo-Ortiz et al. (2010) indicated that light triggered the degradation of PIF1 by photoactivated phytochromes, which allowed *PSY* expression and subsequently rapid production of carotenoids. In addition, the relative concentration of zeaxanthin and violaxanthin in plant photosynthetic tissues is important in stimulating energy dissipation within light-harvesting antenna proteins through non-photochemical quenching to protect against photoinhibition. Under high light condition, violaxanthin is de-epoxidized into zeaxanthin by VDE to dissipate light energy, whereas the reverse reaction converts zeaxanthin to violaxanthin by ZEP under dark condition (Demmig-Adams and Adams, 2002). In conclusion, light played a significant role in regulation of carotenoid biosynthesis in green tissues, but how light ultimately regulates this process remains to be elucidated. Further researches are required to illustrate the carotenoid synthesis regulation in chloroplasts.

Regulatory mechanisms of carotenoid biosynthesis in non-green tissues are distinct from those in green tissues. Briefly, there are three major mechanisms affecting carotenoid accumulation in non-green plant tissues: (1) regulation of genes controlling carotenoid biosynthesis; (2) the regulation of genes for carotenoid degradation; and (3) the regulation of plastid development. Various lines of evidence show that the MEP pathway, GGPP pool, PSY and branch point enzymes might be key regulatory nodes for carotenoid content. They are discussed in detail below.

### Regulation of Isoprenoid Precursor

Carotenoid biosynthesis requires an available source of isoprenoid substrates derived from the MEP pathway, which is a key bottleneck influencing flux through the entire pathway (Farré et al., 2010; Rodriguez-Concepcion, 2010). In the MEP pathway, the transcript levels of DXS, 1-deoxy-D-xylulose 5-phosphate reductoisomerase (DXR) and 1-hydroxy-2-methyl- 2-(*E*)-butenyl 4-diphosphate reductase (HDR) were positively correlated with carotenoid content in maize endosperm (Vallabhaneni and Wurtzel, 2009; Suwarno et al., 2015).

In addition to its role in carotenoid biosynthesis, GGPP is a precursor for synthesis of many other terpenoid compounds in plants. Therefore, the pool of GGPP represents the metabolic link between biosynthesis of carotenoids and other terpenoids, and is responsible for inter-pathway regulation via competition for GGPP. The expression level of GGPP synthase (*GGPPS*) was positively correlated with endosperm carotenoid content in maize (Vallabhaneni and Wurtzel, 2009; Suwarno et al., 2015). Another key regulatory issue is what mechanisms control the partitioning of precursors into various terpenoid pathways. There is clear evidence for multiple *GGPPS* genes in *Arabidopsis*, encoding dedicated enzymes for different branches of various terpenoid pathways (Okada et al., 2000).

### Regulation of Carotenoid Biosynthesis

Phytoene synthase catalyzes the first committed step in carotenoid biosynthesis and is generally accepted as the most important regulatory node in the carotenoid biosynthesis pathway, whose transcripts were positively correlated with carotenoid accumulation (Cong et al., 2009; da Silva Messias et al., 2014). Moreover, PSY seems to be a key integrator for several signals regulating carotenoid biosynthesis. For example, blocking of the MEP pathway and loss-of-function of *PDS* result in down-regulation of *PSY*, whereas increased activity of *DXS* induces *PSY* expression in tomato (Rodriguez-Concepcion et al., 2001; Laule et al., 2003). Orange (OR) protein directly interacts with PSY to regulate carotenoid biosynthesis (Zhou et al., 2015). In addition, carotenoid metabolites also regulate PSY protein level and total carotenoid content (Kachanovsky et al., 2012; Arango et al., 2014). For example, expression of the *PSY* gene is positively up-regulated by ABA and has been associated with pre-harvest sprouting in cereals (Fang et al., 2008; Cazzonelli, 2011).

The cyclization of lycopene has a major role in modulating the β, β/β, ε branch ratio, suggesting that coordination between LCYE and LCYB activities may be necessary for regulation of metabolic flux through different branches of the carotenoid pathway (Cazzonelli et al., 2010; Farré et al., 2011). Over-expression of *LCYB* shifts the balance toward the β, β-branch, whereas over-expression of *LCYE* has the opposite effect (Rosati et al., 2000; D’Ambrosio et al., 2004). However, expression of *PSY1*, *CrtI*, and *LCYB* in transgenic maize endosperm increased β, β/β, ε ratio from 1.2 to 3.5 and also enhanced flux through the β, ε-branch of the pathway, producing almost 25 times more lutein than the normal level (Zhu et al., 2008). Naqvi et al. (2011) also found that when metabolic flux is shifted toward β-carotene there is still enough flux through the β, ε-branch to produce more lutein. These examples showed that regulation of the flux through different branches of the pathway was complex.

Some other carotenogenes also regulated carotenoid content. For example, viviparous mutants *vp5*, *vp2*, and *w3* in maize have defective copies of the *PDS* gene and exhibit increased accumulation of phytoene (Matthews et al., 2003). High expression of the *ZDS* gene was consistent with accumulation of lycopene during carrot root development (Clotault et al., 2008). ZISO and CRTISO are essential for establishing an equilibrium between *cis*- and *trans*- carotenoid isomers (Chen et al., 2010; Yu et al., 2011). In addition, expression of *crtRB1* was negatively correlated with β-carotene levels and positively correlated with zeaxanthin levels in maize (Yan et al., 2010; da Silva Messias et al., 2014).

Apart from the carotenogenes *per se*, transcriptional factors regulating carotenoid biosynthesis have been reported. Reduced transcript level of *RAP2.2*, a member of the APETALA2 (AP2)/ethylene-responsive element-binding protein transcription factor family, was accompanied by a significant decrease in transcript levels of both *PSY* and *PDS* with a concomitant 30% decrease in carotenoid content relative to wild-type (Welsch et al., 2007). The transcription factor RIN induces *PSY1* expression to regulate the flux of carotenoid biosynthesis in tomato (Martel et al., 2011). Moreover, epigenetic regulation was also considered important in carotenogenesis. A chromatin-modifying histone methyltransferase enzyme SDG8 (SET DOMAIN GROUP 8) maintains a transcriptionally permissive chromatin state surrounding the *CRTISO* and thus is able to regulate carotenoid content (Cazzonelli et al., 2009). Overexpression of *microRNA156* in *Brassica napus* enhanced carotenoid content in seeds (Wei et al., 2010).

### Regulation of Carotenoid Degradation

Recent studies have demonstrated that the carotenoid pool is determined in part by the rate of carotenoid degradation (Vallabhaneni and Wurtzel, 2009; Gayen et al., 2015). The expression of *CCD1* or *CCD4* was negatively correlated with carotenoid accumulation (Gonzalez-Jorge et al., 2013; da Silva Messias et al., 2014). It was shown that down-regulation of LOX enzyme activity reduces degradation of carotenoids in Golden Rice suggesting an effective tool to reduce large economic losses of biofortified rice seeds during storage (Gayen et al., 2015). Compared to carotenoid biosynthesis, little is known about the impact of carotenoid degradation on regulation of carotenoid accumulation, and much more work is needed to understand it.

### Regulation of Carotenoid Sequestration

Various studies have shown that carotenoid accumulation is greatly modulated by size, number, and anatomical structure of the plastids in which carotenoid biosynthesis and storage occur. Organelle biogenesis is a major determinant of plastid size and storage compartment number, and affects carotenoid accumulation by providing a larger sink. CHCR (chromoplast-specific carotenoid-associated protein) enhances carotenoid content in high pigment tomato mutants (*hp1*, *hp2*, and *hp3*) due to increased chromoplast number and/or volume (Galpaz et al., 2008; Kilambi et al., 2013). A mutation in the *OR* gene led to differentiation of plastids to chromoplasts causing enhanced carotenoid accumulation in the curds of cauliflower (Lu et al., 2006). A change in chromoplast architecture is associated with carotenoid composition in *Capsicum* fruits (Kilcrease et al., 2013).

Esterification limits degradation of xanthophylls and increases their sequestration within the chromoplast by increased lipophilic properties and integration into lipid-rich plastoglobules (Ariizumi et al., 2014; Mellado-Ortega and Hornero-Méndez, 2016). Moreover, it was suggested that carotenoid accumulation might be correlated with expression of genes influencing lipoprotein components of chromoplast structures, such as plastid-encoded acetyl coenzyme A, carboxylase D and Hsp21 (Neta-Sharir et al., 2005; Barsan et al., 2012; Carvalho et al., 2012).

Although significant progress has been made in understanding carotenoid regulatory mechanisms in plants, several key issues are yet to be addressed. Firstly, very little is known about the global regulatory mechanisms underlying carotenoid metabolism. Cross-talk between carotenoid biosynthesis and other pathways and how interaction responds to plant growth and development and environment remain unclear. Secondly, the molecular nature of regulation of metabolic feedback remains unknown. Finally, research on regulation of carotenoid biosynthesis has mostly focused on model species and such regulatory mechanisms in non-model species are not well documented, hence restricting a detailed understanding of regulation of carotenoid biosynthesis in specific crops.

## Genetic Manipulation of Carotenoid Biosynthesis in Staple Cereals

Maize, rice, and wheat comprise the main foods for human nutrition. However, carotenoid contents in the grains of these crops are usually low. Therefore, breeding staple cereals with high carotenoid content could have a huge impact on human health, without significantly altering current human diets. Such attempts to enhance carotenoid contents or improve carotenoid composition in staple cereals have been made, mainly based on metabolic engineering and marker-assisted breeding as described below.

### Metabolic Engineering

Various metabolic engineering approaches have been made to increase the levels of nutritionally relevant carotenoids in staple cereals and to enable the use of plants as ‘cell factories’ for producing special carotenoids. Amplification of the rate-limiting enzyme with the highest flux control coefficient is the principal target for manipulation. Alternatively, it may be desirable to change the carotenoid composition or extend the classical carotenoid pathway in the tissue of interest.

A breakthrough in metabolic engineering of carotenoids for improved nutritional value of staple crops was achieved in rice, best-known as ‘Golden Rice.’ Here, daffodil *PSY* and *LCYB* genes together with the bacterial *CrtI* were transferred to a *japonica* rice cultivar in which the β-carotene content in the endosperm was 1.6 μg/g of seed dry weight, providing 10–20% of the recommended daily allowance (RDA) of β-carotene (Burkhardt et al., 1997; Ye et al., 2000). Further optimization of the pathway using the maize *PSY* gene driven by a rice glutelin promoter considerably increased carotenoid formation in transgenic rice endosperm, resulting in Golden Rice II lines with carotenoid levels up to 37 μg/g (Paine et al., 2005). Higher carotenoid accumulation was recently achieved through the combined expression of *ZmPSY1*, *PaCRTI* with *AtDXS* or *AtOR* in rice endosperm, suggesting that the supply of isoprenoid precursors and metabolic sink are important rate-limiting steps in carotenoid biosynthesis (Bai et al., 2016). Similarly, total carotenoid levels in wheat were enhanced by co-transformation with maize *PSY1* and the bacterial *CrtI* gene, but the elevation of carotenoid content was only moderate compared with that in the donor wheat cultivar EM12 (Cong et al., 2009). In order to further enrich the provitamin A content in wheat grains, the bacterial *CrtB* and *CrtI* genes were co-transformed into cultivar Bobwhite (Wang et al., 2014), resulting in a total carotenoid content increase to 4.76 μg/g, a β-carotene increase to 3.21 μg/g, and a provitamin A content increase to 3.82 μg/g. Recently, higher levels of β-carotene accumulation up to 5.06 μg/g were obtained by simultaneously overexpressing *CrtB* and silencing carotenoid hydroxylase (Zeng et al., 2015b). Although the level was still insufficient to combat VAD, the progress was still important, as a small increase in carotenoid contents in wheat grains would have a large impact based on the huge daily consumption of wheat-based products throughout the world.

A wide variety of unusual keto-carotenoids and carotenoid intermediates, such as astaxanthin, adonixanthin, 3-hydroxye chinenone, and echinenone have been engineered in transgenic maize plants with seed colors ranging from white and yellow to dark-red, despite the white-endosperm genetic background (Zhu et al., 2008). The carotenoid pathway in rice was recently further extended to form astaxanthin and 4-keto-α-carotene, with co-transformation of *ZmPSY1*, the bacterial *CrtI* and β-carotene ketolase genes (Breitenbach et al., 2014).

As already mentioned, most of the research on carotenoid manipulation in staple cereals has focused on a few main carotenogenes. In the future, manipulation of carotenoid biosynthesis could be extended to different regulatory nodes, such as the MEP pathway, carotenoid degradation, and sequestration. Moreover, the current status of metabolic engineering is somewhat restricted due to its reliance on gene-by-gene approaches. In other pathways, the focus has shifted from individual genes or collections thereof toward overarching regulatory mechanisms that may allow multiple genes in the pathway to be controlled simultaneously. Although enhancement of carotenoid biosynthesis by metabolic engineering proves to be a useful tool, the transgenic lines may induce hitherto undiscovered feedback mechanisms with unpredictable results. One of the major hurdles for commercialization of genetically engineered crops is the legal requirements and acceptance by consumers in various countries. Golden Rice has not yet been released in any country although daily consumption of 75 g of Golden Rice II grains can receive the RDA of β-carotene (Paine et al., 2005).

### Marker-Assisted Breeding

Over the past decade, increasing carotenoid content in grains of staple cereals such as rice, maize, and wheat, has been an important breeding objective. However, conventional breeding to select for QTL with positive effects on carotenoid levels is a slow and laborious process. The identification of rate-limiting steps, the elucidation of molecular basis of known QTL, or the characterization of new alleles for higher carotenoid content, will allow development of functional markers or gene-specific markers for a more efficient selection in breeding. Such functional markers allow breeders to select quantitative traits at the gene level rather than at the phenotypic level.

In maize, previous studies showed that two polymorphic sites within *PSY1* each explained 7 and 8% of the total carotenoid variation (Harjes et al., 2008); four polymorphic sites in *LCYE* explained 58% of β, β/β, ε branch ratio variation and a threefold difference in provitamin A compounds (Yan et al., 2010); three polymorphisms in *crtRB1* were significantly associated with variation in carotenoid content (Fu et al., 2013). Allele-specific markers of three key genes involved in maize endosperm carotenoid biosynthesis were developed to facilitate provitamin A biofortification in maize through marker-assisted selection (MAS). The effectiveness of these molecular markers was verified across diverse tropical yellow maize inbred lines (Azmach et al., 2013; Babu et al., 2013). A favorable *crtRB1* allele was introgressed into seven elite inbred parents using a *crtRB1*-specific marker, and concentration of β-carotene among *crtRB1*-introgressed inbreds varied from 8.6 to 17.5 μg/g, with a maximum increase of up to 12.6-fold over recurrent parent (Muthusamy et al., 2014). Introgression of a favorable allele of the *crtRB1* gene using molecular markers also significantly increased provitamin A content in quality protein maize inbred lines (Liu et al., 2015). In rice, no carotenoids were detected in the endosperm due to lack of endosperm-specific *PSY* expression (Ye et al., 2000). Therefore, molecular marker-assisted breeding for rice carotenoid improvement is still not feasible. Although many molecular markers have been developed for genes involved in carotenoid biosynthesis in wheat as described below, there are no reports of higher carotenoid content wheat cultivars developed by marker-assisted breeding.

The objectives of Harvest Plus^2^, a worldwide collaboration that drives biofortification as a project within the Consultative Group of International Agricultural Research (CGIAR), are to breed more nutritious cultivars of staple food crops by conventional breeding technologies strengthened with molecular markers. Provitamin A-biofortified crops, including maize, cassava, and sweet potato, have been developed and released in Nigeria, Zambia, and Uganda. Eating orange sweet potato has been shown to improve vitamin A status of children.

The carotenoid biosynthesis is very complex, therefore multiple genes must be taken into consideration during marker-assisted breeding in order to enhance the accuracy of prediction and selection. In addition, mutants with desirable carotenogenic properties generated by chemical treatment may provide new insights into carotenoid improvement in staple cereals that are not categorized as genetic manipulation and can be immediately introduced into breeding programs. Meanwhile, such mutants are not involved in the expensive and time-consuming gene transformation, and therefore, easy to be used in breeding programs.

## Carotenoids in *Triticum spp.*

Carotenoids, the main components of grain yellow pigment in wheat determine the flour color and affect both the nutritional value of the grain and its utility in different applications (Mares and Campbell, 2001). High yellow pigment is a very important quality parameter for pasta made from durum wheat and yellow alkaline noodles made from bread wheat, but low or medium levels of yellow pigment are preferred for Chinese white noodles and steamed bread produced by bread wheat. Thus, manipulations of yellow pigment in opposite directions are important breeding objectives in bread wheat and durum breeding programs. However, compared with maize and rice, carotenoid biosynthesis in wheat has received much less attention. Therefore, we provide a comprehensive overview of carotenoid biosynthesis in wheat in order to facilitate future studies of the carotenoid metabolism.

### Carotenoid Profiles in Wheat

Lutein is the predominant carotenoid in wheat, and accounts for 80–90% of total carotenoids along with small amounts of zeaxanthin, α-carotene, β-cryptoxanthin, and β-carotene (Abdel-Aal et al., 2007; Digesù et al., 2009). The pigments are variably distributed in the seed; the endosperm has the highest lutein content, whereas zeaxanthin and β-carotene are concentrated near the outer layers of the kernel (Hentschel et al., 2002; Borrelli et al., 2008). Although levels of carotenoids in wheat are low, there is significant genetic variation. Previous studies showed that primitive and wild relatives, landraces, and synthetic hexaploids usually accumulate higher levels of carotenoids. For example, einkorn (2*n* = 14), and Khorasan and durum wheat (2*n* = 28) contain higher levels of lutein (5.4–7.4 μg/g) compared to common wheat (1.9 μg/g; Hidalgo et al., 2006).

Carotenoid biosynthesis during grain development was examined using a doubled haploid (DH) bread wheat population (Howitt et al., 2009). During the early stages of grain development, carotenoids from the β, β-branch (zeaxanthin, antheraxanthin, and violaxanthin) were present at higher levels than those from the β, ε-branch (lutein). The highest amounts of lutein and zeaxanthin were detected at 10 days post anthesis (DPA). Although the level of lutein did not change significantly during endosperm development, carotenoids from the β, β-branch declined gradually and were undetectable in mature grains.

### QTL Underpinning Carotenoids in Wheat

Although environmental factors play an important role in determining carotenoid contents in wheat, the genetic component is predominant and heritability is relatively high at 0.85–0.97 for YPC, a trait strictly related to carotenoids (Elouafi et al., 2001; Van Hung and Hatcher, 2011).

The genetic architecture of YPC was investigated through QTL analysis in both durum and bread wheat. QTL located in the telomeric regions of the long arms of the homeologous group 7 chromosomes, especially 7AL and 7BL, largely influenced YPC (Elouafi et al., 2001; Patil et al., 2008). Various minor QTLs were also detected on chromosomes of homeologous groups 2, 3 and 4, and chromosomes 1A, 1B, 5A, 5B, 6A, and 6B (Zhang et al., 2008; Blanco et al., 2011; Colasuonno et al., 2014). In addition, the 1BL.1RS wheat-rye translocation carried a major QTL for YPC and b^∗^ explaining 25.4–32.2% of the phenotypic variance (Zhang et al., 2009; Zhai et al., 2016). Wheat cultivars with the 1BL.1RS translocation had higher total carotenoid contents (0.76 vs. 0.61 μg/g), lutein (0.46 vs. 0.40), zeaxanthin (0.08 vs. 0.07) and β-carotene (0.22 vs. 0.14) than those without the translocation (Li et al., 2016), an aspect that should be considered in breeding for higher provitamin A content in bread wheat.

### Gene Cloning and Molecular Marker Development

Most of carotenogenes in wheat have been cloned and characterized. Briefly, the full-length genomic DNA sequence of *PSY1* was cloned, and two co-dominant markers (*YP7A* and *YP7B-1*) and two dominant markers (*YP7B-2* and *YP7B-3*) were developed for *PSY-A1* and *PSY-B1* (He et al., 2008, 2009). *YP7A* co-segregated with a QTL for YPC on chromosome 7AL and explained 20–28% of the phenotypic variance (He et al., 2008). Cultivars with *PSY-B1c* had the highest YPC (2.01 μg/g), followed by *PSY-B1a* (1.71 μg/g), whereas those with *PSY-B1b* had the lowest value (1.40 μg/g; He et al., 2009).

Dong (2011) cloned the full-length *PDS* gene and designed two complementary markers *YP4B-1* and *YP4B-2* corresponding to higher and lower YPC, respectively (no significant difference). The full-length genomic sequence of *ZDS* was cloned and co-dominant molecular markers *YP2A-1* and *YP2D-1* were developed for *ZDS-A1* and *ZDS-D1*, respectively (Zhang et al., 2011; Dong et al., 2012). *YP2A-1* and *YP2D-1* co-segregated with QTL for YPC on chromosome 2A and 2DL, respectively, explaining 11.3–18.4% of the phenotypic variance.

The entire sequence of the *LCYE* gene was isolated and located on homoeologous group 3 chromosomes, and it was identified as a candidate gene underlying QTL for lutein content on chromosome 3B (Howitt et al., 2009). Dong (2011) developed a co-dominant functional marker *YP3B-1* for *TaLCYE-B1*, but values of YPC from cultivars with *TaLCYE-B1a* were not significantly different from those with *TaLCYE-B1b*. Therefore, the effect of *TaLCYE-B1* on carotenoid contents in wheat grains need to be further investigated. *e-LCY3A-3*, a co-dominant functional marker, was developed based on *e-LYC3Aa* and *e-LYC3Ab* alleles (Crawford and Francki, 2013b). A highly significant (*P* < 0.01) association with QTL on chromosome 3A indicated that *e-LYC3A* is functionally associated with variation in b^∗^. The *TaLCYB* gene was cloned and shown to have a role in β-carotene biosynthesis using RNAi (Zeng et al., 2015a). In addition, *HYD1*, *HYD2*, and *HYE* were cloned and characterized (Kawaura et al., 2009; Qin et al., 2012). Information relating to these genes and molecular markers is provided in **Table 1.** The functional markers have been used in routine germplasm characterization and cultivar development.

**Table 1:** Summary of carotenogenic genes and molecular markers in bread wheat.

Enzyme	Gene	GenBank No.	Chromosomal location (IWGSC)	Marker	Allele	Fragment size (bp)	YPC
Phytoene synthase 1	*PSY1*	EF600063	7AL, 7BL,7DL	*YP7A*	*PSY-A1a/PSY-A1c*	194	High
					*PSY-A1b*	213	Low
				*YP7B-1*	*PSY-B1a*	151	Medium
					*PSY-B1b*	156	Low
				*YP7B-2*	*PSY-B1c*	428	High
				*YP7B-3*	*PSY-B1d*	884	–
Phytoene desaturase	*PDS*	FJ517553	4AS, 4BL, 4DL	*YP4B-1*	*TaPDS-B1b*	562	High
				*YP4B-2*	*TaPDS-B1a*	382	Low
ζ-Carotene desaturase	*ZDS*	HQ703016	2AS, 2BS, 2DS	*YP2A-1*	*TaZDS-A1a*	183	Low
					*TaZDS-A1b*	179	High
				*YP2D-1*	*TaZDS-D1a*	No	High
					*TaZDS-D1b*	981	Low
Lycopene ε-cyclase	*LCYE*	EU649785	3AL, 3B, 3DL	*e-LCY3A-3*	*e-LCY3Aa*	537	–
					*e-LCY3Ab*	309 & 230	
				*YP3B-1*	*TaLCYE-B1a*	635	–
					*TaLCYE-B1b*	No	
Lycopene β-cyclase	*LCYB*	FJ814767	6AS, 6DS				
Carotenoid β-ring hydroxylase	*CHYB1*	JX171673	2AL, 2BL, 2DL				
	*CHYB2*	JX171670	6AL, 6BL, 6DL				
Carotenoid ε-ring hydroxylase	*CHYE*	AK334877	1AL, 1BL, 1DL				


*IWGSC, International Wheat Genome Sequencing Consortium; YPC, yellow pigment content; – Unknown.*

For carotenoid degradation, three copies of the *LOX-1* gene (*LOX-B1.1*, *LOX-B1.2*, and *LOX-B1.3*) were cloned in durum wheat (Hessler et al., 2002; Verlotta et al., 2010). In bread wheat, the full-length genomic DNA sequence of *TaLOX-B1* gene was cloned, and complementary markers *LOX16* and *LOX18* were developed (Geng et al., 2012). However, *CCD* sequences of wheat have not been reported to date.

### The Molecular Basis of QTL for Carotenoid Content

With carotenogenes identified and functional markers developed, there is a growing interest in understanding the molecular basis of QTL underpin carotenoid content in wheat.

As expected, *PSY1* gene was considered as a candidate gene responsible for YPC variation in wheat grains since *YP7A* and *YP7B* co-segregated with QTL for YPC on chromosomes 7AL and 7BL (He et al., 2008; Zhang and Dubcovsky, 2008; Singh et al., 2009). Other studies indicated that a second gene other than *PSY1* in the distal regions of chromosomes 7A and 7B affects YPC (Singh et al., 2009; Crawford and Francki, 2013a). The geranylgeranyl transferase I α-subunit (*RGGT*) gene was mapped to distal regions on chromosomes 7BL and 7DL (Crawford et al., 2008). This gene encodes enzyme involved in the terpenoid backbone biosynthesis pathway, providing the precursor GGPP for carotenoid biosynthesis, and it could be a candidate for the additional gene. Moreover, a *Cat3-A1* gene was co-located to the QTL for b^∗^ on 7AL, encoding a catalase enzyme which controls varying degrees of bleaching action on lutein by regulating hydrogen peroxide accumulation in developing wheat grain, and it could be another candidate for the additional gene (Crawford and Francki, 2013a; Li et al., 2015).

The *LCYE* gene was considered as a candidate gene for QTL affecting b^∗^ variation and lutein content on chromosomes 3A and 3B in bread wheat (Howitt et al., 2009; Crawford and Francki, 2013b). In addition, a QTL for pasta color on chromosome 4B was linked to a polymorphic deletion in *LOX-B1*, suggesting that it was associated with pigment degradation during pasta processing (Hessler et al., 2002).

With advances in genomics and bioinformatics, some other genes were found to be associated with carotenoid biosynthesis in wheat. A genome scan for QTL in durum and SNP homology prediction against annotated proteins in the wheat and *Brachypodium* genomes identified diphosphomevalonate decar boxylase (*DMAPD*) and aldehyde oxidase (*AO*) co-located with the major QTL for YPC on chromosomes 5BL and 7AL, respectively (Colasuonno et al., 2014). Six candidate genes related to terpenoid backbone biosynthesis were within QTL intervals associated with four color-related traits in bread wheat (Zhai et al., 2016); these included genes for alpha-1,3-mannosyl-glycoprotein 2-beta-*N*-acetylglucosaminyltransferase (*MGAT1*), mevalonate kinase (*MK*), delta-cadinene synthase (*DCS*), ketol-acid reductoisomerase (*KARI*), cytokinin oxidase/dehydrogenase (*CKX*), and secologanin synthase (*SLC*). All these genes further enrich carotenoid biosynthesis pathway (**Figure 1**).

Because quantification of carotenoids by HPLC is expensive and time-consuming, most studies of wheat carotenoid contents have depended on indirect parameters such as YPC and b^∗^. In order to deepen understanding of the carotenoid metabolism in wheat, fast, cost-effective methods to detect individual carotenoids should be developed and improved, such as UPLC (ultra-high performance liquid chromatography), UPLC-MS and UPLC-NMR. Moreover, many QTLs affecting carotenoid content could not be explained by known genes. This provides opportunities to discover additional genes controlling carotenogenesis in wheat grain. With progress in next-generation DNA sequencing and SNP chips, it will be much easier to construct high-density genetic maps useful in detecting QTL for carotenoid content, identifying candidate genes, and map-based cloning of candidate genes.

## Future Prospects

As discussed above, significant progress has been made in our understanding of carotenoid metabolism, genetic regulation, and genetic manipulation in higher plants. This has improved our capacity for breeding new cultivars with high carotenoid contents. Compared to other plants, there are still numerous unknown aspects on carotenoid biosynthesis in the staple cereals. Firstly, a more comprehensive and deeper understanding of carotenoid regulatory mechanisms will undoubtedly facilitate genetic manipulation to modify overall carotenoid contents and individual components with predictable outcomes. Secondly, genetic manipulations in crops were mainly focused on β-carotene enhancement to combat the VAD, but improvements in other carotenoids were rarely reported, even for lutein and zeaxanthin which play significant roles in promoting eye and skin health and in reducing the risk of several chronic diseases. Therefore, future studies should give more attention to improve other carotenoids or simultaneously engineer multiple carotenoid molecules. In addition, the carotenoid pathways in maize and rice have been extended to accumulate a wide variety of unusual keto-carotenoids, which could be exploited to other crop plants, including wheat.

New technologies provide novel opportunities for genetic manipulation of carotenoid biosynthesis in staple cereals. With progress in next-generation DNA sequencing and SNP chips, genomic selection is expected to play a key role in breeding programs (Varshney et al., 2014). KASP (Kompetitive Allele Specific PCR) technology with its much faster and higher detection accuracy offers cost-effective and scalable flexibility in application of gene-specific markers in breeding programs (Semagn et al., 2014). Development of practical breeding chips based on KASP markers and closely linked SNP markers from GWAS will be a big step forward in improving marker application in breeding high provitamin A-enriched cereals. New gene editing technologies, such as TALENs (transcription activator-like effector nucleases) and CRISPR (clustered regularly spaced palindromic repeat), are currently the most widely used methods for understanding gene function, and are emerging as transformative technologies for crop breeding due to ability to edit genomic sequences at defined sites rather than random introduction of foreign DNA (LaFountaine et al., 2015). We are strongly confident that provitamin A-enriched crops will be developed in the near future by application of improved genetic knowledge and new technologies.

## Author Contributions

SZ wrote the paper. XX and ZH designed and wrote the paper.

## Conflict of Interest Statement

The authors declare that the research was conducted in the absence of any commercial or financial relationships that could be construed as a potential conflict of interest.

## Footnotes

1 http://www.who.int/nutrition/topics/vad/en/.

2 http://www.harvestplus.org/
